# A Family‐Centred Paediatric Delirium Bundle: A Feasibility Study

**DOI:** 10.1111/nicc.70103

**Published:** 2025-06-29

**Authors:** Rikke Louise Stenkjaer, Ingrid Egerod, Mala Moszkowicz, Gorm Greisen, Janne Weis, Erwin Ista, Jakob Gjedsted, Marianne Steen Jensen, Kirsten Krone Reichl, Suzanne Forsyth Herling

**Affiliations:** ^1^ Department of Neonatal and Pediatric Intensive Care Copenhagen University Hospital Rigshospitalet Copenhagen Denmark; ^2^ Department of Intensive Care Copenhagen University Hospital Rigshospitalet Copenhagen Denmark; ^3^ Department of Clinical Medicine University of Copenhagen Copenhagen Denmark; ^4^ Child and Adolescent Mental Health Center, Copenhagen University Hospital – Mental Health Services CPH Hellerup Denmark; ^5^ Department of Pediatric Surgery Pediatric Intensive Care, Erasmus MC – Sophia Children's Hospital Rotterdam the Netherlands; ^6^ Department of Cardiothoracic Anesthesia Copenhagen University Hospital Rigshospitalet Copenhagen Denmark; ^7^ Department of Pediatric Cardiology Copenhagen University Hospital Rigshospitalet Copenhagen Denmark; ^8^ The Neuroscience Centre, Copenhagen University Hospital Rigshospitalet Copenhagen Denmark

**Keywords:** delirium, family‐centered care, feasibility study, non‐pharmacological, paediatric intensive care unit

## Abstract

**Background:**

Paediatric delirium (PD) is a common disorder in critically ill children. PD management, however, needs improvement. To this end, we developed a family‐centred non‐pharmacological delirium management bundle based on consensus from paediatric intensive care unit experts and parents. The bundle included interventions such as developing a day structure and encouraging parent presence.

**Aim:**

We aimed to test the feasibility of our PD bundle by investigating acceptability, implementation and practicality for parents, nurses and physicians.

**Study Design:**

A multiple‐method process evaluation study with quantitative and qualitative data, using questionnaires and focus group interviews. To determine the feasibility of the PD bundle, we set provisional goals for the acceptability, implementation and practicality prior to conducting our study. The study took place in two paediatric cardiac units from March to May 2024.

**Results:**

Two focus group interviews were held with 15 nurses and four physicians. Parents, nurses and physicians found the PD bundle acceptable because all the interventions were intuitively relevant and familiar, meeting our target goals to proceed with an RCT study. We surveyed 31 parents to estimate the degree of implementation of the 11 interventions in the PD bundle. Six of the interventions in the bundle were delivered in more than 80% reported by the parents, while five fell short of our target goals. Nurses and physicians suggested that the PD bundle could be implemented if the interventions were consolidated into fewer elements. We trained 90% of the invited nurses and physicians to deliver the PD bundle, which met our target goal of 80%. Hospital organisation, environment and differences in hygienic policy interpretations challenged the practical application of the PD bundle according to the nurses and physicians.

**Conclusions:**

The PD bundle appears promising for acceptance, implementation and practical application in clinical practice. However, as the delivery of the PD bundle fell short of our target goals, a revised PD bundle should be tested before performing an RCT.

**Relevance to Clinical Practice:**

A non‐pharmacological paediatric delirium bundle is now available to critical care nurses to prevent and manage delirium in critically ill children.

**Summary**

- What is known about the topic
  ○ Paediatric delirium is a common disorder in critically ill children.
  ○ Solid evidence of the effects of pharmacological drugs on the prevention and management is lacking.
  ○ Non‐pharmacological interventions may be efficacious in reducing the incidence and duration of delirium.
- What this paper adds
  ○ A family‐centred paediatric non‐pharmacological delirium bundle is feasible for use in the PICU.
  ○ Parents, nurses and physicians stress the importance of parental engagement in delirium management.
  ○ The use of the PD bundle increases awareness of delirium medication and delirium assessments.

## 1 Background

Paediatric delirium (PD) is a common disorder in critically ill children affecting attention, cognition and awareness and is associated with poor outcomes [1, 2, 3]. Several validated PD‐screening tools are available and are increasingly being implemented in paediatric intensive care units (PICU) [4, 5, 6, 7]. While screening tools enable the identification of PD, the question of optimal PD management remains.

Solid evidence of the effects of pharmacological drugs on the prevention and treatment of PD is lacking [8, 9]. A few studies report on the pharmacologic management of PD using antipsychotic medications, but studies show that side effects such as extrapyramidal symptoms and prolonged QT interval are common [10, 11, 12].

Non‐pharmacological interventions in adult intensive care unit (ICU) patients may be efficacious in reducing the incidence and duration of delirium [13, 14, 15], but little research has been conducted in the paediatric setting [16, 17, 18]. Based on paediatric literature, the Society of Critical Care Medicine suggested non‐pharmacologic strategies for the management of PD, such as improved sleep hygiene, interdisciplinary rounds with family engagement, early mobilisation and family involvement with direct childcare [19]. Recently, we conducted a Delphi study with a panel of international PD experts to investigate the most important interventions to be included in a non‐pharmacologic delirium management bundle for the PICU. Our proposed PD bundle included three overall domains: supporting cognition, supporting sleep and physical activity [20]. The PD bundle is evidence‐based with the intention of informing healthcare professionals of best practices while considering clinical expertise and parent/child preferences [21].

Parent engagement in the non‐pharmacological management of PD is important [20]. Based on parents' views on the PD bundle, we found that the parents agreed that most of the suggested interventions were relevant but cautioned that the bundle should not be administered too rigidly [22].

Ultimately, we plan to conduct a randomised controlled trial (RCT) to test the benefits and harms of the PD bundle. In preparation for this study, we need to ensure that the trial can be successfully executed in a busy PICU. This feasibility study may identify the need for intervention modifications and changes before testing the effect in an RCT setup. Parents, nurses and physicians need to understand how to apply the bundle, and intervention fidelity should be satisfactorily identified as the degree to which an intervention is delivered as intended [23].

## 2 Aim

The aim of the study was to test the feasibility of our PD bundle by investigating (1) acceptability, (2) implementation and (3) practicality for parents, nurses and physicians.

## 3 Methods

### 3.1 Design

We conducted a multiple‐method process evaluation study, with quantitative and qualitative data, using questionnaires and focus group interviews. To allow reproducibility and transparency, we employed elements of Bowen et al.'s framework for assessing feasibility [24]. The framework lists eight general focus areas, some relevant for particular intervention phases depending on the outcome of interest [24]. Table 1 provides an overview of the aspects of feasibility, data collection methods and analysis methods used in this study.

**TABLE 1:** Overview of focus areas and goals for feasibility.

Area of focus for feasibility and main question	Aspects of feasibility	Methods of data collection	Methods of data analysis	Goals to proceed with an RCT study
Acceptability				
To what extent is the bundle judged as suitable, satisfying or meaningful to deliver?	Perceived appropriateness	Focus group interviews—two groups of a mix of nurses and physicians	Qualitative content analysis	A general perception of appropriateness with an overweight of facilitators rather than barriers
Implementation				
To what extent can the bundle successfully be delivered to critically ill children?	Degree of execution Degree of recruitment	Data from questionnaires answered by parents and registration sheets	Descriptive statistics (frequencies) Descriptive statistics (frequencies)	80% of each intervention is delivered, defined by the parents 80% of the families should wish to participate in the study, with a maximum dropout rate of 5%
Practicality				
To what extent can the bundle be carried out with intended participants using existing resources and circumstances?	The ability of participants to carry out intervention activities Instruction of the bundle	Focus group interviews – two groups of a mix of nurses and physicians	Qualitative content analysis Descriptive statistics (frequencies)	A general perception of appropriateness with an overweight of facilitators rather than barriers 80% of all invited clinicians should receive instruction

### 3.2 Setting

The study was conducted at two adjacent paediatric units: cardiac intensive care (five beds) and semi‐intensive care (nine beds) at a University Hospital in Copenhagen. We wished to test our PD bundle in this setting because these children are at particular risk for PD [19, 25, 26]. Most rooms in the units are double occupancy, and few are single rooms. Both units have free visiting hours for the parents, but at the semi‐intensive care unit, a parent is expected to be with the child 24 h a day. Both units use the principles of family‐centred care (FCC) according to policy. These two units are the most specialised units of their kind in Denmark, providing treatment and care for children with medical and surgical heart diseases.

### 3.3 Population and Sampling

We included parents of children aged 0–17 years admitted for elective cardiac surgery. The intervention started at the child's admission and ended at hospital discharge. Parents were contacted by the primary investigator (PI) on the day of admission prior to the child's surgery to offer study information. The parents were informed verbally and in writing about the aim of the study and expected parental participation. We used a consecutive sample identified by the coordinating cardiac nurse, who identified and recorded potential parents of children for elective cardiac surgery. Since the literature offers no clear guidance on sample size for feasibility studies, we intended to include a minimum of 20 parents in the inclusion period available to us from March to May 2024.

All nurses (Registered and Vocational) and physicians at the two units using the PD bundle were included as participants in this study. The management team at the two units selected the focus group participants. Verbal and written information on the aim of the focus group was provided.

### 3.4 The PD Bundle

The non‐pharmacologic delirium management bundle consisting of 11 generic interventions was developed for critically ill children 17 years of age or younger (Table S1) [20]. The bundle was based on consensus among international PICU experts [20]. The interventions are similar to adult delirium recommendations [27], such as developing a daily structure, adjusting light exposure according to the time of day, scheduling time for sleep and providing eyeglasses and hearing aids if appropriate. The PD management bundle should be delivered by nurses and physicians with family engagement and individualised to the child's age, parent/child preferences and family needs according to parents' perspectives [22].

### 3.5 Introducing the Study

Unit management in both units endorsed the study and allocated resources for training, engagement of key persons and time for implementing the PD bundle. Key persons were selected, including two or three nurses and one physician at each unit. The role of the key persons was to ensure focus, motivation and training while reducing potential barriers. Posters depicting the bundled interventions were placed at the workstations and patient rooms. Demographic and clinical data for each child were recorded during the study from March to May 2024.

### 3.6 Training of Nurses and Physicians

The PI conducted nurse and physician training in using the PD bundle. The training sessions consisted of three elements: (1) Introduction to the PD bundle, (2) the practicality of the 11 interventions and (3) discussion of the intervention. During training, it was stressed that some of the interventions would influence the work routines of all nurses and physicians and would demand flexibility in carrying out procedures and medical rounds according to the child's needs and the elements in the bundle. A certain ‘mindset’ is recommended before the staff enters the patient room, focusing on communication and appropriate voice level. We assumed that the bedside nurses would have a central role in collaboration with the parents and that the intervention would be delivered inter‐professionally to be successful. We expected the PD bundle would disturb daily work routines and therefore allowed time for discussion before and during implementation. During the training sessions, the nurses and physicians suggested that the PD bundle be across all age groups instead of three different age‐group bundles to ease the implementation. The PI stated that the interventions should be offered individually and age‐appropriately. The non‐pharmacological PD bundle poster can be found in the Figure S1.

### 3.7 The Questionnaire

We developed a questionnaire to assess parental experience of the implementation of the bundle. The questionnaire was based on the 11 interventions in the PD bundle and rephrased as yes/no questions for each of the 11 items to assess whether the bundle was used as intended, for example, “Did the staff speak calmly and clearly to your child?” or “Did the staff develop a day structure for your child in collaboration with you?” The parents had the opportunity to add narrative answers and comments at the end of the questionnaire.

### 3.8 Focus Groups

Two focus group interviews were conducted to explore the nurses' and physicians' experiences using the PD bundle from each unit. Interviews took place at the hospital and lasted about an hour. The PI moderated both focus groups using a semi‐structured interview guide (Table S2). The observer was a child psychiatrist who recorded non‐verbal responses in her field notes. Both the PI and the observer were known to the participants during the training sessions and previous PD studies. The PI ensured that all participants had ample opportunity to express their views and attitudes. The interviews were audio‐recorded and transcribed verbatim by the PI.

### 3.9 Data Analysis

The quantitative data regarding the questionnaires and patient demographics were analysed using descriptive statistics. The qualitative data consisted of the narrative responses from the questionnaires and the transcription of the focus groups. They were analysed deductively using qualitative content analysis [28, 29]. We used the three analytical phases proposed by Elo and Kyngäs: preparation, organising and reporting [28]. During the preparation phase, we coded each source (questionnaire and focus group interview) as a unit of analysis focusing on the manifest content. The moderator and observer read the transcripts several times to immerse themselves in the data. During the organising phase, we developed a structured case‐by‐category matrix based on each sampling approach and coded deductively according to the predefined categories of acceptability, implementation and practicality. The three predefined categories are described in Table 1. Codes and categories were discussed in the author group. We pursued credibility by investigator triangulation, transferability by describing participants and context, dependability by providing quotes and confirmability by describing the sampling, data collection and analysis [30]. The coding and organisation of data were assisted by the computer software NVivo version 14 [31]. The consolidated criteria for reporting qualitative research guidelines were used to ensure the transparency of our qualitative reporting (Table S3) [32].

### 3.10 Feasibility Evaluation

To determine the feasibility of the PD bundle, we set provisional goals for the acceptability, implementation and practicality prior to conducting this study (Table 1).

### 3.11 Ethical Considerations

This study was approved by the National Committee on Health Research Ethics: H‐23076858 (February 29th 2024), the two management teams and the Danish Data Protection Agency (j.no.: P‐2019‐548). All included families and staff were informed about the study by the PI, received written information and provided written consent to participate.

## 4 Results

### 4.1 Demographics of Parents, Nurses and Physicians

All parents of children who met the inclusion criteria gave consent to participate. We included 31 parents. None of the parents dropped out. Seven parents were excluded due to postponement of surgery (*n* = 5) or inability to speak Danish (*n* = 2). The degree of recruitment (100%) and no dropouts met our target goal of 80% participation with a maximum dropout rate of 5% (Table 1). The focus group interviews with nine and 10 participants, respectively, were held in the respective units. The participants included four physicians and 15 nurses, two male and 17 female, with 5–39 years of work experience with an interquartile range of 26.5 years. The mean duration of the interviews was 50 min, and the attendance rate was 100%. Table 2 shows the demographics of the children of participating parents.

**TABLE 2:** Demographic data of included children (*n* = 31).

Characteristics children	Number (%)
Gender	
Female	19 (61)
Male	12 (39)
Age categories of included children	
0–2 years	20 (65)
3–5 years	5 (16)
6–17 years	6 (19)
Length of mechanical ventilation in days, mean (range)	1 (1–3)
Length of PICU stay in days, mean (range)	3 (2–6)
Length of hospital stay in days, mean (range)	8 (6–12)
Admission diagnosis	
Ventricular septal defect (DQ210)	8 (26)
Atrial septal defect (DQ211)	6 (19)
Steno‐fallot tetralogy (DQ213)	4 (13)
Atrioventricular deptal defect (DQ212)	3 (10)
Vascular ring of aorta (DQ254I)	2 (6)
Other cardiac diagnosis	8 (26)

### 4.2 Training

We planned eight training sessions for 58 nurses and physicians. The attendance rate was 90%, with 43 nurses and nine physicians. In addition, students and interested collaborators attended (*n* = 6). We met our target goals, where 80% of all invited clinicians should receive instruction on the PD bundle (Table 1).

### 4.3 Degree of Execution of the PD Bundle

All included parents (*n* = 31) returned the completed questionnaire, and 22 (22/31) provided narrative responses. Six of the 11 interventions were delivered in more than 80% of cases reported by the parents. Thereby, five interventions from the PD bundle fell short of our target goals. Responses on bundle elements are shown in Table 3. The parents experienced that the hospital staff spoke calmly and clearly, developed a daily structure in collaboration with the parents, provided appropriate lighting, avoided loud talking and dimed light using curtains or blinds. None of the children required eyeglasses or hearing aids.

**TABLE 3:** Responses to survey items (*n* = 31).

Question	Number of yes (%)
Did the staff speak calmly and clearly to your child?	31 (100)
Did the staff develop a day structure for your child in collaboration with you?	25 (81)
Did the staff provide appropriate lighting according to the time of the day?	30 (97)
Did the staff ensure your child using eyeglasses and hearing aids if appropriate?	31 (100)
Did the staff encourage you to be present in the patient room?	13 (42)
Did the staff encourage the presence of familiar objects around your child's bed?	19 (61)
Did the staff schedule time for sleep for your child?	9 (29)
Did the staff provide sleep objects from home or play music to support sleep?	17 (55)
Did the staff avoid loud talking in the child's room?	29 (94)
Did the staff dim the light using curtains or blinds?	28 (90)
Did the staff document and evaluate daily mobilisation goals?	13 (42)

*Note:* Staff: Nurses and physicians.

### 4.4 Qualitative Findings

The main categories and sub‐categories are shown in Table 4. In the following, we denote nurses as N, physicians as P and parents jointly as PA, followed by the study number.

**TABLE 4:** Main categories and sub‐categories.

Main categories	Sub‐categories
Acceptability	The interventions are intuitively relevant The interventions are familiar
Implementation	The interventions can be consolidated The presentation of the bundle can be improved
Practicality	Increasing the visibility of the bundle Benefits of the bundle. Challenges to the bundle

#### 4.4.1 Acceptability (Main Category)

##### 4.4.1.1 The Interventions Are Intuitively Relevant (Sub‐Category)

The nurses and physicians considered the interventions in the bundle important to their children or their work, although they were not written in a guideline, policy or procedure. The unwritten interventions were described as intuitive ways to collaborate with the parents and care for the child—and as part of their professional training. One parent described the bundle as an improvement to clinical practice focusing on the specific child's needs and the active parental role in direct child care.

N6 “Yes, some of the interventions are second nature although they are not put into writing. Sometimes, if I enter a room at 10:30 pm and find the light and TV on, it's natural to act. I also did this before.”

P1 “…but it makes a big difference if it's NOT done, and it makes a big positive difference if it's DONE.”

##### 4.4.1.2 The Interventions Are Familiar (Sub‐Category)

The nurses and physicians agreed that the interventions could be successfully delivered because they were already a part of daily practice and had been for several years. The interventions were essential but not new to them.

N15 “I must say that I find it hard to understand because this is what we have always done, although it was not written on paper.”

#### 4.4.2 Implementation (Main Category)

##### 4.4.2.1 The Interventions Can Be Consolidated (Sub‐Category)

The informants identified overlapping interventions that might present a barrier to implementation. Examples of overlapping interventions were: (1) ‘speak calmly and clearly’ and ‘avoid loud talking’; (2) ‘provide appropriate lighting according to the time of the day’ and ‘dim light using curtains or blinds.’ The informants agreed that eyeglasses and hearing aids were out of place in the bundle because they were too apparent. They suggested combining the overlapping interventions to increase the usability of the bundle. This would enable the visual presentation of the bundle with larger pictograms and font size for clarity.

N2 “I actually think that they could be reduced to fewer [interventions] as many interventions are connected.”

##### 4.4.2.2 The Presentation of the Bundle Can Be Improved (Sub‐Category)

The informants discussed ideas for further implementation of the bundle, such as introducing it as standard to all new employees, preparing pre‐printed schedules for circadian rhythm and mobilisation and presenting it to parents during the pre‐operating meeting. Incorporating routines and schedules in daily practice was suggested to enhance implementation by giving awareness and structure to the nurses and physicians themselves, as well as to interprofessional partners and parents.

N2 “This is something we talked about today, that there are things we can work with. Continue this project. It has some good points. We also talked about making a schedule for physiotherapists, so they can record mobilisation of older children or make a reward system or day/night schedule. We also talked about a pre‐printed schedule to complete with the patient.”

#### 4.4.3 Practicality (Main Category)

Nurses and physicians reflected on the bundle's practical application, with particular attention to the interventions, benefits and spin‐offs leading to reduced use of [delirium] medications. Structural challenges, such as the hospital as a system, the capacity of rooms and hygiene demands, were noted.

##### 4.4.3.1 Increasing Visibility of the Bundle (Sub‐Category)

This sub‐category describes how to best display and encourage dialogue about the interventions. The posters depicting the 11 interventions were placed at workstations and patient rooms to prompt dialogue and remind staff to adhere to the bundle. Some nurses applauded the intervention because it reduced daily squabbles, while other nurses were affronted, being reminded to provide normal nursing care.

N9 “What I like about the [bundle posters] is that we have not had the same staff discussions since we got the posters. I like it because we all have been in a trial period. The visibility has prevented some of the arguments we had.”

The posters also served as a reminder to introduce the bundle to the parents, allowing them to understand the interventions and initiate action. Parents appreciated the visibility of the interventions, which enabled them to help promote a new culture in the unit.

N2 “I have used it [the poster] a few times. I hung them up in all patient rooms. I don't know if you have used them, but I actually think it's a good talking paper for the parents about our expectations of each other. It shows how [the parents] can help their child during the admission.”

##### 4.4.3.2 Benefits of the Bundle (Sub‐Category)

Two main benefits of the bundle were discussed. One was increasing the focus on PD and pharmacological/non‐pharmacological management, and the second was articulating the degree of parent engagement.

The bundle was designed to promote non‐pharmacological PD management and prevention. According to the physicians, the bundle prompted reflection on medications as a spin‐off. They appreciated the non‐pharmacological aspect of delirium management in the bundle. Moreover, the nurses focused more on delirium assessment than before.

P1 “It has meant a lot in more than 10‐20‐30 situations where medications were discussed because we also discussed this [the non‐pharmacological bundle]. It helps us to think and wait in situations where we eventually avoid using medications. We might even be able to see a reduction in sedatives now.”

N3 “We discuss [delirium assessment] more. Well, I think he or she assessed delirium, and then we put the child by the mother, and then the SOS‐PD score was reduced, I think. We have had more focus on this.”

The bundle was designed to promote parent engagement. Nurses, physicians and parents agreed that it is logical for parents to deliver many of the interventions described in the bundle. The professionals emphasised the importance of informing and educating parents to foster an empowering culture that allows parents to deliver the interventions. Parents naturally felt they should be involved as they considered they had better knowledge of their child.

PA26 “We have not experienced a special focus on these interventions by the staff. We can't say that the interventions have been ignored. We have followed the interventions as a natural part of our stay.”

#### 4.4.4 Challenges to the Bundle (Sub‐Category)

Several factors, including hospital organisation and interprofessional collaboration, physical surroundings and differing interpretations of hygienic policy, challenged the execution of the bundle.

The nurses described competing agendas within the hospital and agreed that the interventions depended on the time available for patient care and coordination with external staff, such as X‐ray techs and housekeeping. When the unit was busy, keeping promises made according to the bundle was unfeasible. The physicians tried to adhere to the bundle, but it was more hassle and time‐consuming, and rounds could take close to a whole day rather than a few hours.

N3 “…and other times when we are expecting x‐rays, blood draws or other – even if we have agreed on 10–12 o'clock or something to ensure rest – the rest of the hospital doesn't know this. Then we can't keep our promise to the patient, even if it says ‘rest’ on the door.”

Adherence to the PD bundle was challenged by the lack of space and noise in double rooms with two critically ill children, their equipment, their parents and their staff. Single rooms were prioritised whenever feasible and were highly valued by the parents.

N2 “We can have so many good intentions about giving rest and protecting the child, but when the other patient is screaming, it does not work. The physical surroundings make it difficult, but I think our focus changes when we have more time and are able to move the patients around to provide single rooms when possible.”

Differing interpretations of the hygienic policy challenged the bundle. Practice regarding the use of stuffed animals from home varied not only in the two units but also among nurses in the same unit.

N11 “The staff disagrees; some say no; they can have the polar bear we give out, which is sterile.”

Some interventions in the bundle were impractical due to the physical surroundings, for example, dimming the light with curtains or blinds appeared straightforward. Still, it was challenging due to the absence of curtains that could reduce the early morning sunlight.

PA19 “Blackout curtains are missing, particularly in the morning, because it gets light early.”

## 5 Discussion

In this feasibility study, we tested the acceptability, implementation, and practicality of our PD bundle. We set a priori goals to determine feasibility and ensure the transparency of the data interpretation.

Nurses and physicians found the PD bundle acceptable because the interventions were relevant and well known from FCC. The parents experienced that the interventions improved practice. Although the interventions were familiar to the nurses, the parents did not report universal application.

It is well known that FCC, which has similarities to our bundle, is challenging to implement [33]. This might be due to the theory‐practice gap where patient care is subjective and often depends on the nurses' interpretation, self‐boundaries and active participation [33]. It is known that groups of professionals can share blind spots that limit change and innovation [34]. We believe that implementing FCC requires a culture change with support from the management team to continuously articulate the FCC elements and describe how FCC can be practiced in each unit.

Our results suggest that the PD bundle can be implemented in a PICU, but the nurses and physicians recommended improvements on clarity and avoidance of overlapping issues. As might be expected, the single rooms provided more opportunities to employ the bundled interventions than double rooms. Single‐ rooms are associated with greater family satisfaction and lower delirium prevalence than multi‐occupation rooms. Staff members, however, might find single rooms more stressful due to obstruction caused by family presence and less communication with colleagues [35]. This is a central discussion, as the nurses and physicians may experience that the quality of their care is compromised in single rooms. There is a trend, however, to build children's hospitals with single rooms, and consensus is that nurses adapt to working in the new environment [36].

Parent engagement is an essential part of the PD bundle. The nurses and physicians stressed the importance of informing parents to foster an empowering culture and encouraging them to deliver the interventions. This agrees with the literature stating that parents wish to participate in care if informed and supported by the nurses [37, 38]. In our study, the parents were already with their child during the PICU stay and delivered interventions such as bringing familiar objects from home. Parents wish to be involved in delirium management with particular tasks to focus on [22]. The parents in our study, however, indicated that their presence was not always encouraged by the staff. A poorly formulated question regarding family presence may be understood as advice not to leave the patient room. Policies regarding parental presence varied in the two units. Across different countries, PICUs have different visitation, presence and participation policies, but family presence is important to critically ill children [39].

The PD bundle benefited practice by increasing awareness of PD assessments among nurses and discussions of medications among physicians. Although the interventions in the bundle can be seen as common sense, they are core elements of the prevention and management of PD [18]. Drugs like opioids, benzodiazepines and steroids are independent risk factors for delirium, and studies show that regular PD assessments are not always performed [40, 41]. If implementing our PD bundle encourages reflection on medication use and increases PD assessments, it will benefit the children.

Our PD bundle was intended as an evidence‐based guideline for practice, but like all guidelines, individualisation is necessary at the patient and unit levels. As an example, we found that the two units in our study had different policies on bringing stuffed animals from home due to different hygienic policies according to whether the child came directly from the operating room or was in a step‐down unit focusing on recovery and activity. The idea of bringing a stuffed animal from home is to support sleep and cognition because the smell and texture can evoke strong feelings, memories and comfort. A stuffed animal from the hospital may be a substitute in cases where the toy from home presents an infection risk to the child.

The delivery of the PD bundle fell short of our target goals since only half of the interventions were applied to the children according to the participating parents (Table 3). A revision of the PD bundle according to the nurses' and physicians' suggestions should be pilot‐tested before conducting an RCT. We recommend a cluster RCT to reduce contamination of the control group.

## 6 Strengths and Limitations

The combination of qualitative and quantitative data strengthened our study. Another strength was that we tested the feasibility of two different types of units. Nevertheless, the study also has limitations. This was a single‐centre study with a small sample of cardiac children. A stronger design would be a multi‐centre investigation with a mixed PICU population. Also, the questionnaire was not tested before its application. The majority of parents responding to the questionnaire were parents of children younger than 2 years of age. Therefore, older children are underrepresented, and we may be missing some insights. Both units were using the principles of FCC before implementing the PD bundle. Implementing the PD bundle into PICUs without the knowledge of FCC might cause more barriers than experienced in this study.

## 7 Implications and Recommendations for Practice

Critical care nurses are now provided with a non‐pharmacological PD bundle that is acceptable and practical in clinical practice to prevent and manage PD. The PD bundle is a low‐tech, low‐cost and simple intervention that increases awareness of PD. Many of the interventions can and should be performed by parents. This will benefit care in a busy PICU while increasing patient comfort by providing a familiar person with the critically ill child.

## 8 Conclusion

In this study, we assessed the feasibility of our PD bundle by investigating acceptability, implementation and practicality for nurses, physicians and parents. The nurses and physicians found the bundle acceptable, as the interventions were described as familiar and relevant. Implementation needed improvement. The PD bundle should be simplified, and the visual presentation should be expanded to improve the PD bundle. A revision of the bundle according to our findings should be discussed in a panel of an interprofessional healthcare group from the hospital together with children and their parents. As delivery of the intervention fell short of our target goals, a revised PD bundle should be tested before performing an RCT.

## Ethics Statement

This study was approved by the National Committee on Health Research Ethics: H‐23076858 (February 29th 2024), the two management teams and the Danish Data Protection Agency (j.no.: P‐2019‐548).

## Consent

All included families and staff were informed about the study by the PI, received written information and provided written consent to participate.

## Conflicts of Interest

The authors declare no conflicts of interest.

## Supporting information


**Data S1.** Supporting Information.

## Data Availability

The data that support the findings of this study are available on request from the corresponding author. The data are not publicly available due to privacy or ethical restrictions.
