# Supplementary material for: A Family‐Centred Paediatric Delirium Bundle: A Feasibility Study
Source: Nurs Crit Care. 2025 Jun 29;30(4):e70103. doi: 10.1111/nicc.70103 (PMC12206954; doi:10.1111/nicc.70103)

# **Supplemental**

# **Table 1 Non-Pharmacologic Delirium Management Bundle-Pediatric Intensive Care Unit (NDB-PICU) by Age Group^a^**

| **Age group**  **0 to 2 years** | **Age group**  **3 to 5 years** | **Age group**  **6 to 18 years** |
| --- | --- | --- |
| **Support cognition** | **Support cognition** | **Support cognition** |
| Speak calmly and clearly |  |  |
|  | Explain who you are when you approach the child. Tell your name to the child | Explain who you are when you approach the child. Tell your name to the child |
| Develop a day structure in collaboration with parents | | |
| Provide appropriate lighting according to the time of day | | |
| Ensure that the child uses eyeglasses and hearing aids if appropriate when awake, and ensure that the glasses are clean, and batteries are working | | |
| Encourage the parents to be present | | |
| Encourage presence of familiar objects around the bed | Encourage presence of familiar objects around the bed |  |
|  |  | Encourage the child to do activities that they liked to do at home (e.g., watch television, use iPad) |
| **Support sleep** | **Support sleep** | **Support sleep** |
| Schedule time for sleep. Ask the parents about the usual sleep rhythm | | |
| Provide sleep objects from home such as teddy bear, sleeping pillow, or cuddle cloth | Provide sleep objects from home such as teddy bear, sleeping pillow, or cuddle cloth |  |
|  |  | Play music according to the child’s preferences. Consult parents |
| Avoid loud talking in the child’s room | Avoid loud talking in the child’s room |  |
|  |  | Close the door if the staff is near to reduce noise |
| Dim light by using curtains or blinds |  |  |
|  | Dim or turn off the artificial light around the child | Dim or turn off the artificial light around the child |
| **Support physical activity** | **Support physical activity** | **Support physical activity** |
| Document and evaluate daily mobilization goals | Document and evaluate daily mobilization goals |  |
|  |  | Make activity goals visible in the child’s room |

^a^ Interventions with the highest rank from all the strategies

Interventions highlighted with grey is similar between all age groups

# **Table 2 Interview guide**

| **Interview guide** |
| --- |
| What are your experiences with the 11 interventions in the PD management bundle? |
| What do you think of the possibility of implementing the 11 interventions in a busy clinical everyday life? |
| How much do these interventions differ from your practice before the project? |
| What could be the reason why some of the interventions are not carried out? |
| To what extent do you think the families have been able to recognize the interventions? |
| Do you think that the 11 interventions are good as a principle? Why/why not? |
| Do you think it makes sense to continue with the 11 interventions? |
| Do you have experience with the 11 interventions that you want to share with us? |

PD: Pediatric delirium

**Table 3:** Consolidated criteria for reporting qualitative research checklist

**
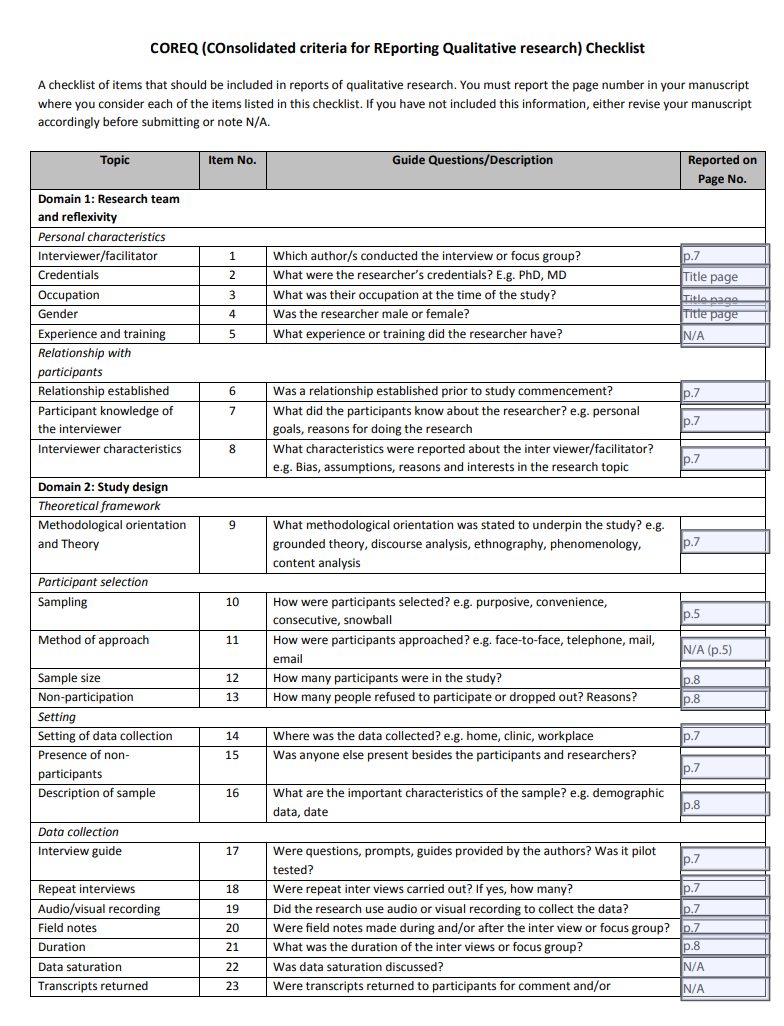
**

**
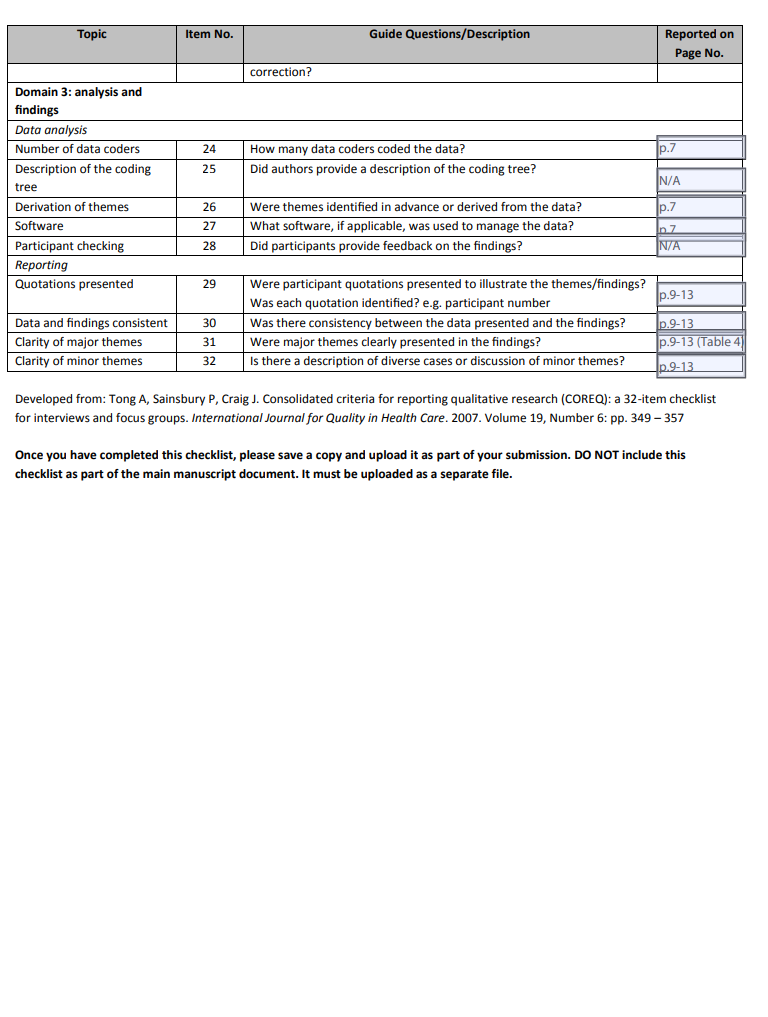
**

**Figure 1: The non-pharmacological PD bundle poster**


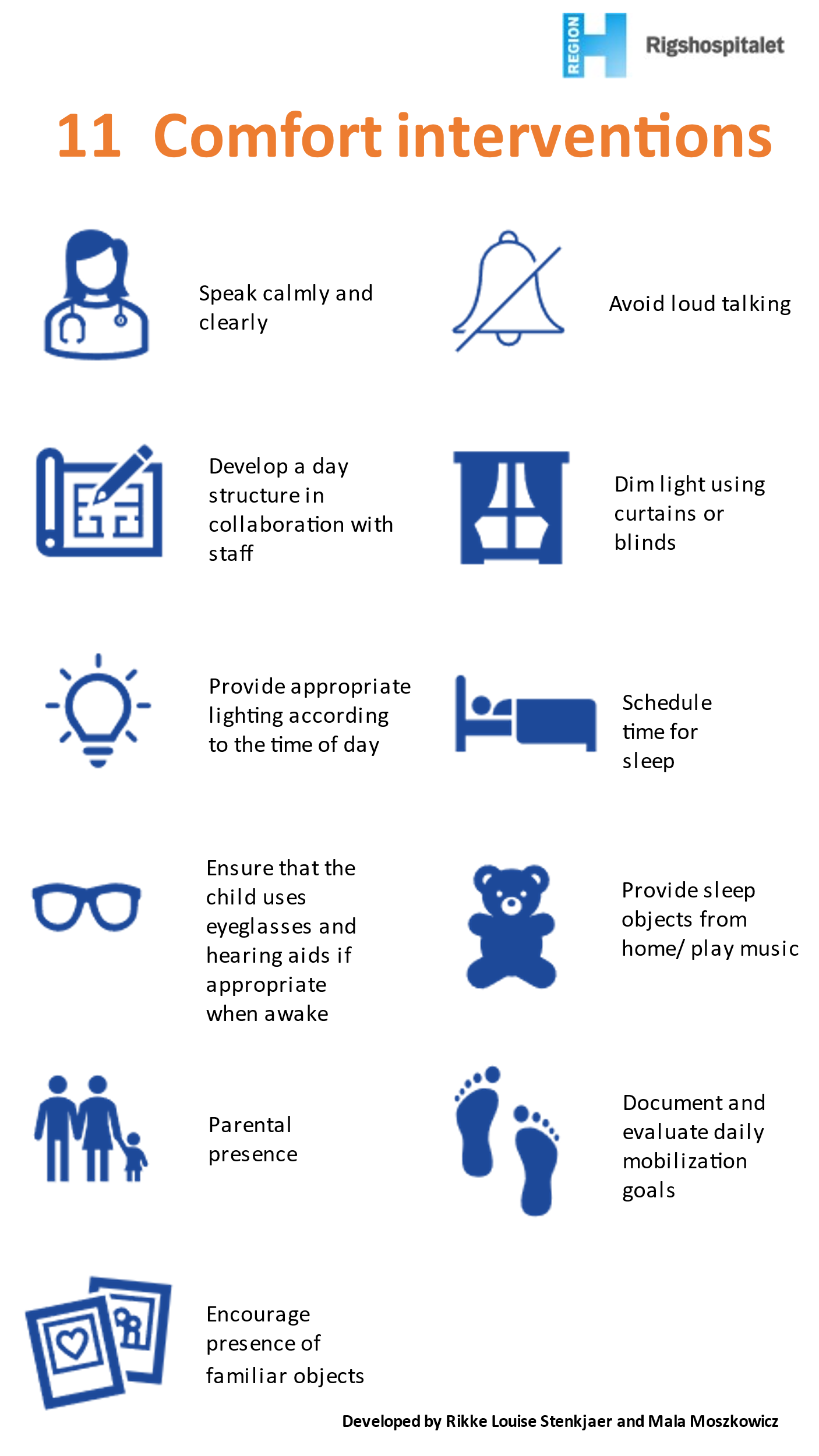

Supplement: Supplementary file 1 — Data S1. Supporting Information. [file NICC-30-0-s001.docx]
